# Supplementary material for: Airway Extracellular Copper Concentrations Increase with Age and Are Associated with Oxidative Stress Independent of Disease State: A Case-Control Study Including Patients with Asthma and COPD
Source: Antioxidants (Basel). 2025 Aug 17;14(8):1006. doi: 10.3390/antiox14081006 (PMC12382632; doi:10.3390/antiox14081006)
Supplement: Supplementary file 1 [file antioxidants-14-01006-s001.zip › antioxidants-3757949-supplementary.pdf]

Supplemental table S1. Inclusion and exclusion criteria for the young groups.

INCLUSION CRITERIA, YOUNG GROUPS

Asthmatics:

- Positive skin prick test against birch pollen or timothy grass.
- FEV_1 > 80% of predicted.
- Methacholine-test PC20 < 8mg/ml.

Non-asthmatics:

- No history of allergy or asthma.
- FEV_1 > 80% of predicted.

EXCLUSION CRITERIA, YOUNG GROUPS

- Age <18 or >40 years.
- Current smoking or history of smoking.
- Airway infection within six weeks prior to or during study.
- Current use of other medication than short-term β2-agonists, with a minimum period of 3 months since last use of inhaled corticosteroids.
- Current antioxidant supplementation.

Supplemental table S2. Inclusion and exclusion criteria for the aged groups.

INCLUSION CRITERIA, AGED GROUPS

COPD current smokers:

- FEV_1 between 30-80 % of predicted.
- $FEV_1/FVC < 0.7$.
- Current smoking with smoking history > 10 pack years.

COPD ex-smokers:

- FEV_1 between 30-80 % of predicted.
- $FEV_1/FVC < 0.7$.
- Current smoking with smoking history > 10 pack years and smoking cessation > 5 years prior to inclusion.

Non-COPD current smokers:

- Normal lung function.
- Current smoking with smoking history > 10 pack years.

Non-COPD never-smokers:

- Normal lung function.
- No history of smoking.

EXCLUSION CRITERIA, AGED GROUPS

- Age <50 and >75 years.
- Asthma, diabetes, cardiovascular disease or other significant disease.
- Airway infection within 6 weeks of bronchoscopic exam or during study.
- Treatment with inhaled corticosteroids (during the last 4 weeks), long-acting bronchodilators (during the last two weeks) and oral steroids (during the last 4 weeks).

- Current use of antioxidant supplementation.
- Pathological findings on ECG and/or blood tests prior to bronchoscopy.
- Pathological findings on chest X-ray (not performed on never-smokers).
- Absence of informed consent.

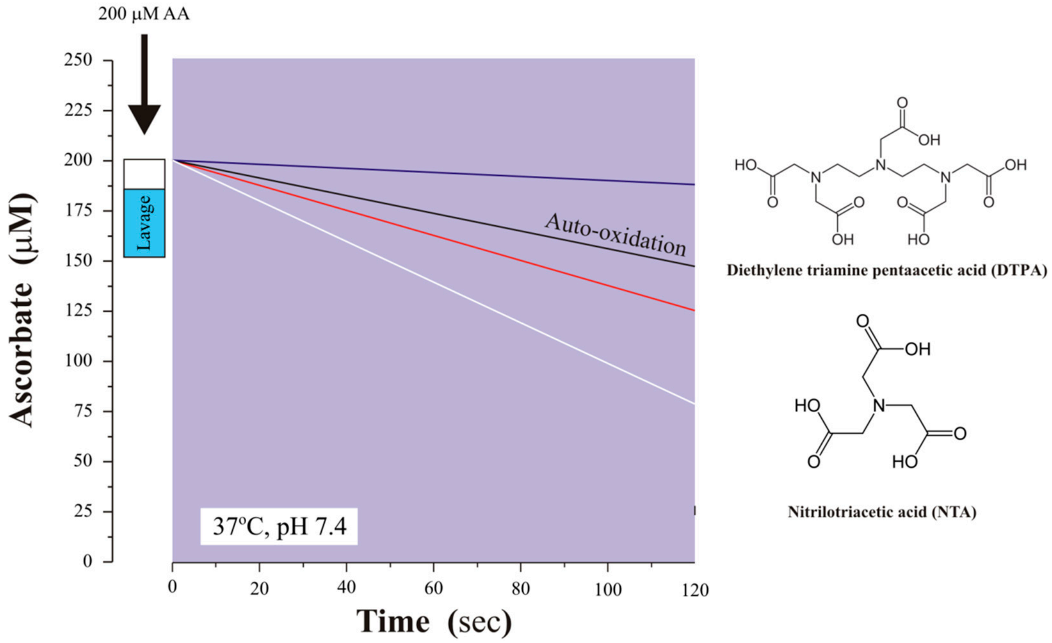

Figure S1. A cartoon illustrating the principle of the ascorbate depletion assay. Lavage samples are spike with ascorbate to achieve a starting concentration of 200 μM. The loss of ascorbate is then monitored at 265 nm every 2 minute for 2 hours. A typical profile for a lavage sample is shown above using the red line. All incubations are also performed parallel to a water blank, which accounts for the background rate of auto-oxidation occurring due to trace metal contamination - black line. Addition of the chelator DTPA in excess (200 μM) should completely abolish any metal dependent catalysis, including that contributing to the background oxidation – purple line. Co incubation conversely may act to accelerate the oxidation of ascorbate, specifically in relation to the formation of Fe-NTA, as it is able to mobilise Fe from other biologic chelators in the biological media – white line.

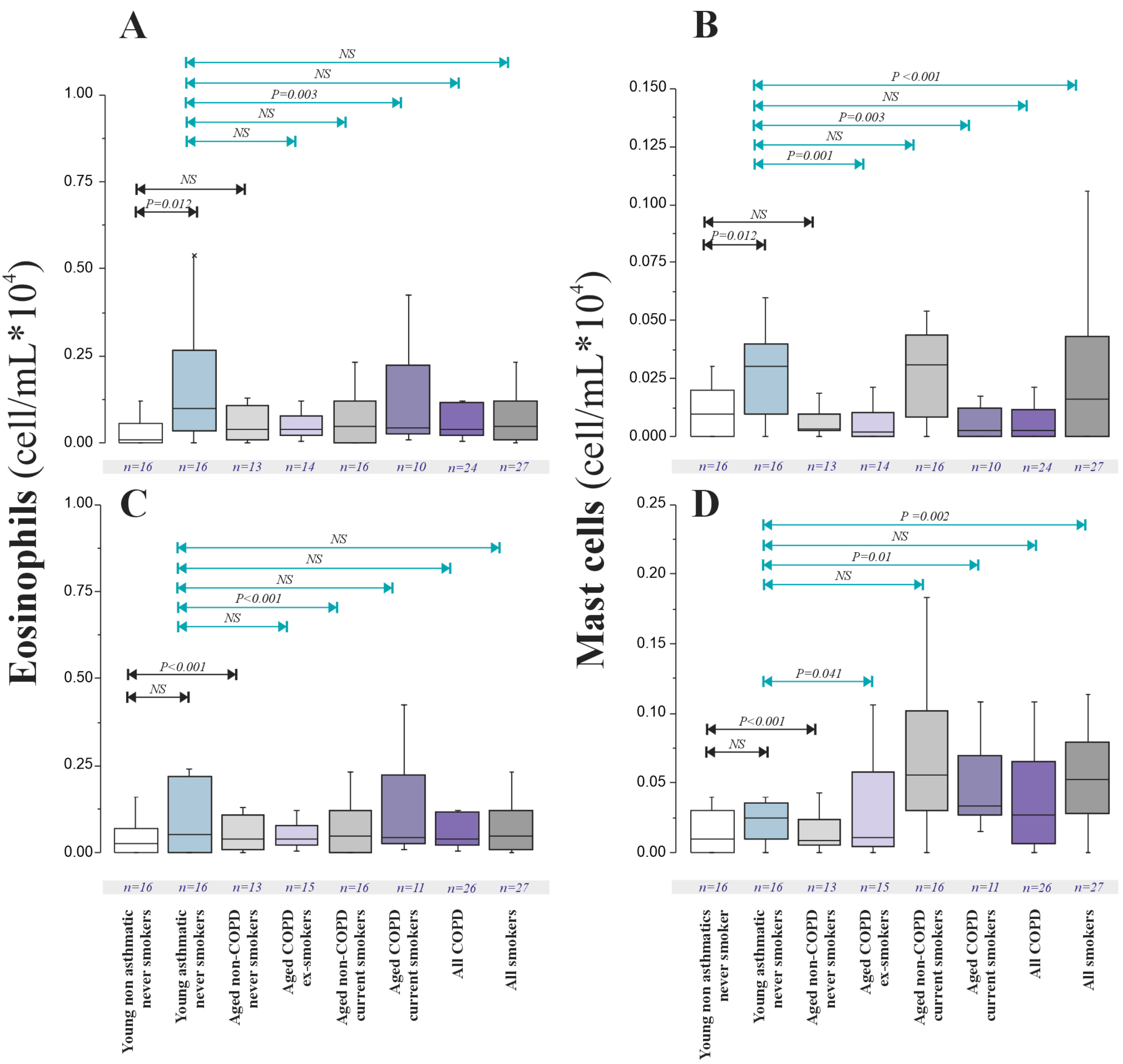

Figure S2. Eosinophil and mast cell numbers measured in BW (A-B) and BAL (C-D) fluid. Recovered from young and aged adults (smokers and non-smokers), asthmatic never-smokers and COPD patients (current smokers and ex-smokers). NS = no significant difference between the indicated groups.
